# Supplementary material for: Bacterial Community Structure and Potential Microbial Coexistence Mechanism Associated with Three Halophytes Adapting to the Extremely Hypersaline Environment
Source: Microorganisms. 2022 May 30;10(6):1124. doi: 10.3390/microorganisms10061124 (PMC9228163; doi:10.3390/microorganisms10061124)
Supplement: Supplementary file 1 [file microorganisms-10-01124-s001.zip › Supplementary figures.pdf]

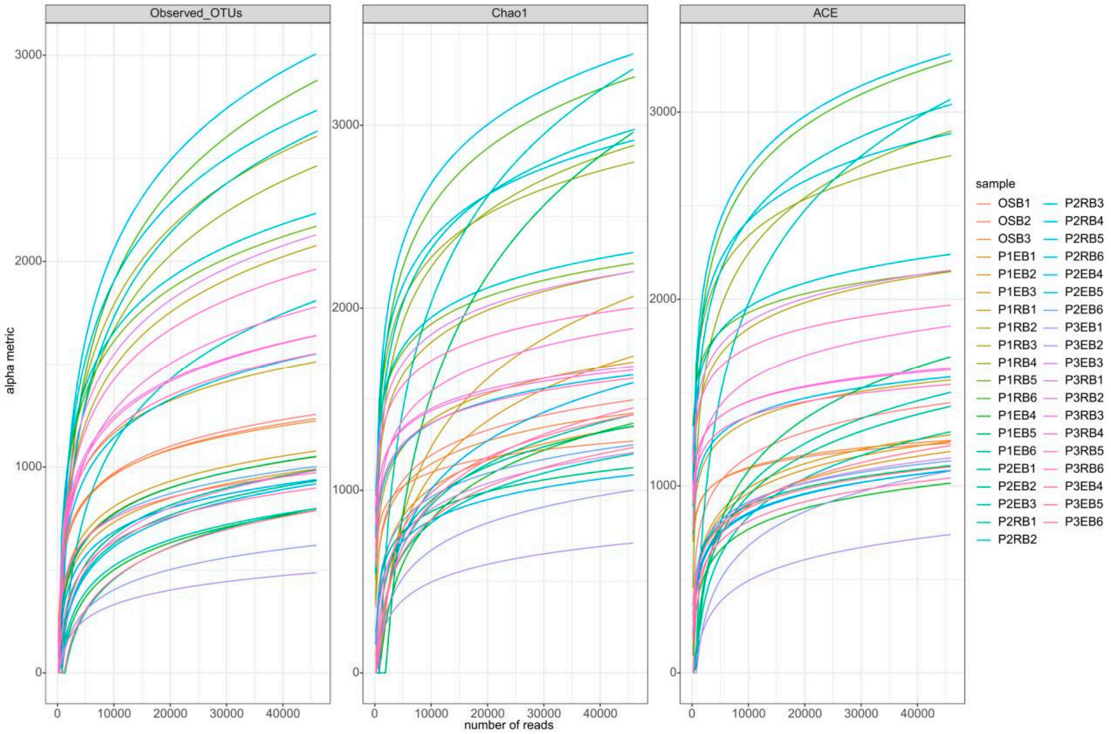

Supplementary Figure S1: Rarefaction curves are based on the sequences of the V3 and V4 hypervariable regions of the 16S rRNA gene from all samples.

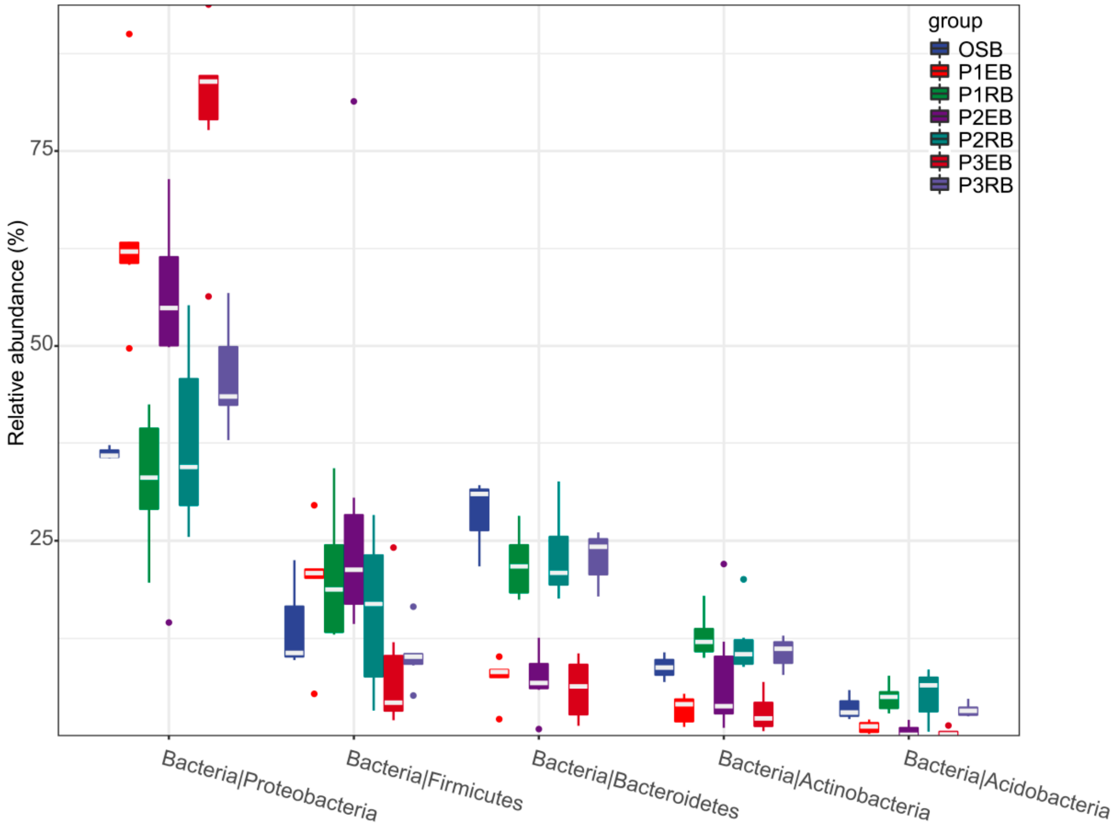

Supplementary Figure S2: The relative abundance of top five bacterial phylum in different sample groups.

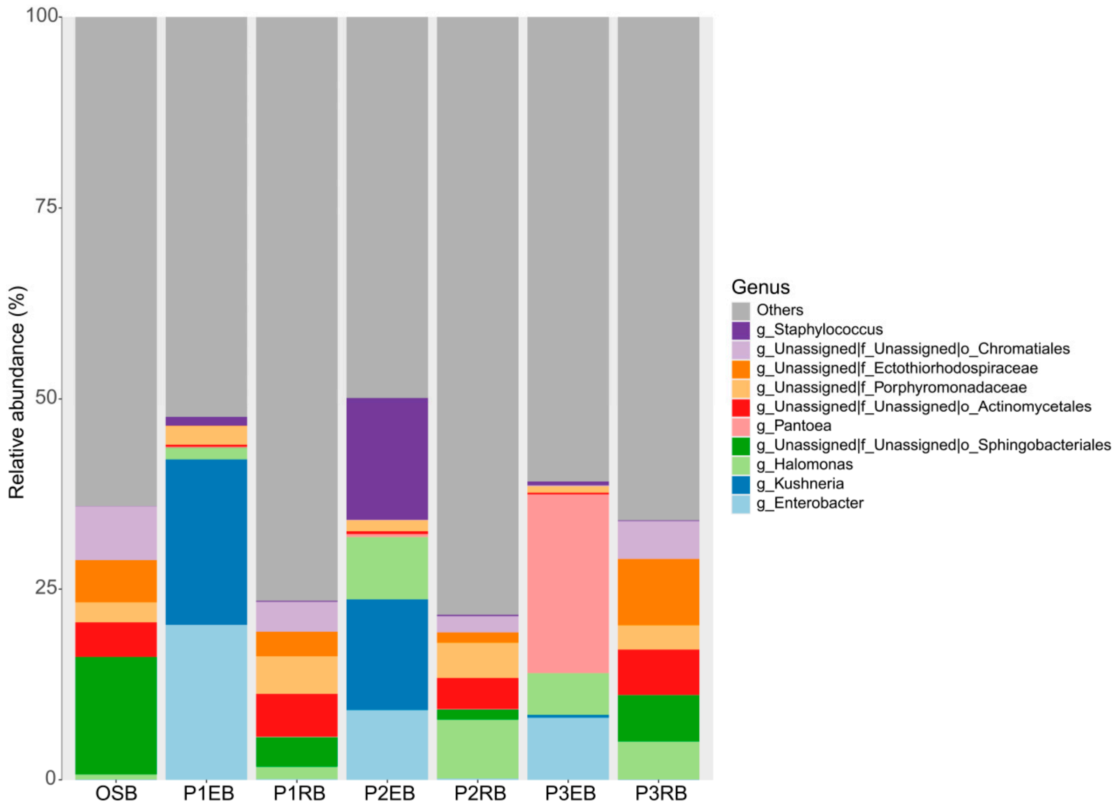

Supplementary Figure S3: Relative abundances of endophytic bacteria and rhizospheric bacteria at the genus level (top ten) in different samples.

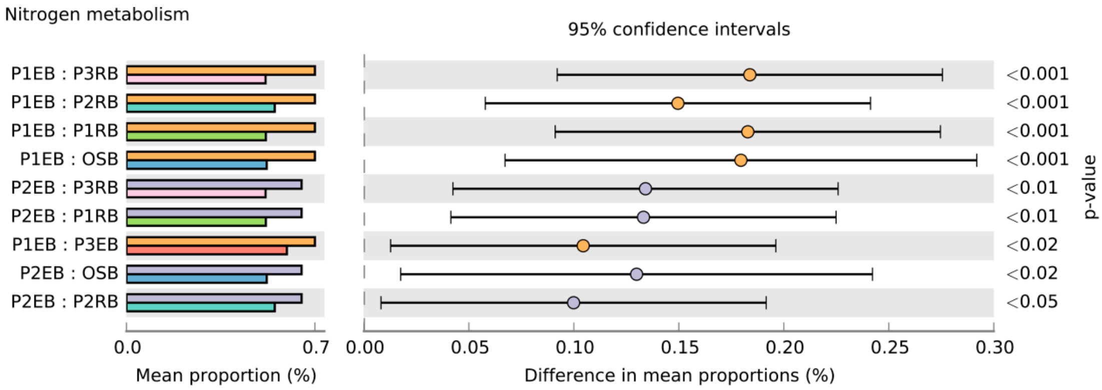

Supplementary Figure S4: The functional abundance difference of Nitrogen Metabolism based on the PICRUSt 2 algorithm to make inferences from KEGG annotated

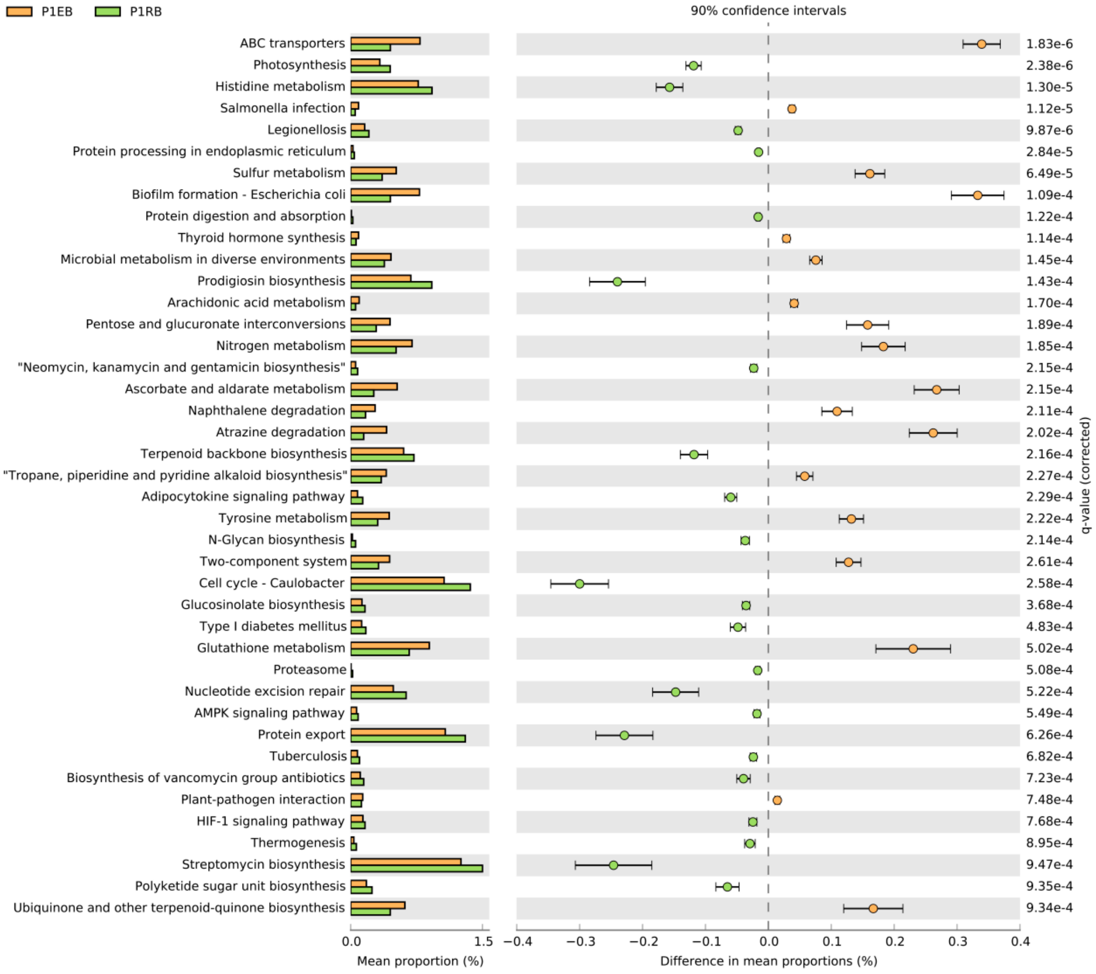

Supplementary Figure S5: The abundance difference of KEGG pathways between the P1EB group and the P1RB group predicted by PICRUSt2.

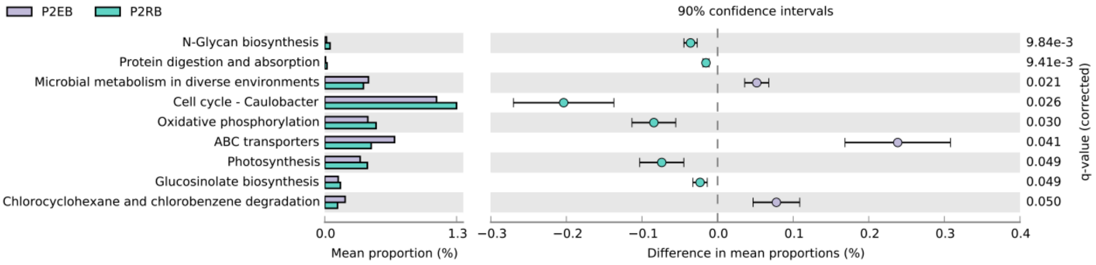

Supplementary Figure S6: The abundance difference of KEGG pathways between the P2EB group and the P2RB group predicted by PICRUSt2.

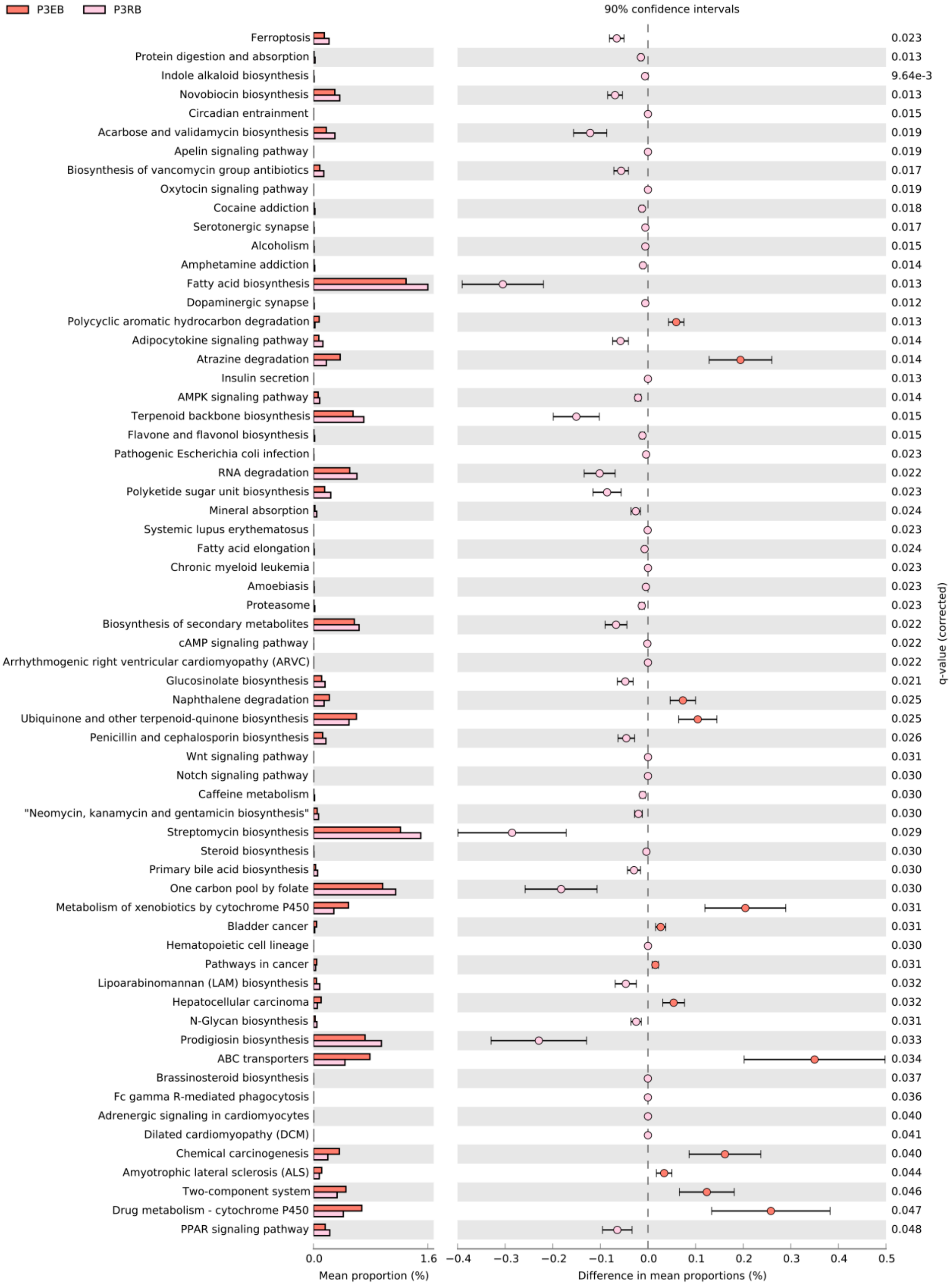

Supplementary Figure S7: The abundance difference of KEGG pathways between the P3EB group and the P3RB group predicted by PICRUSt2.

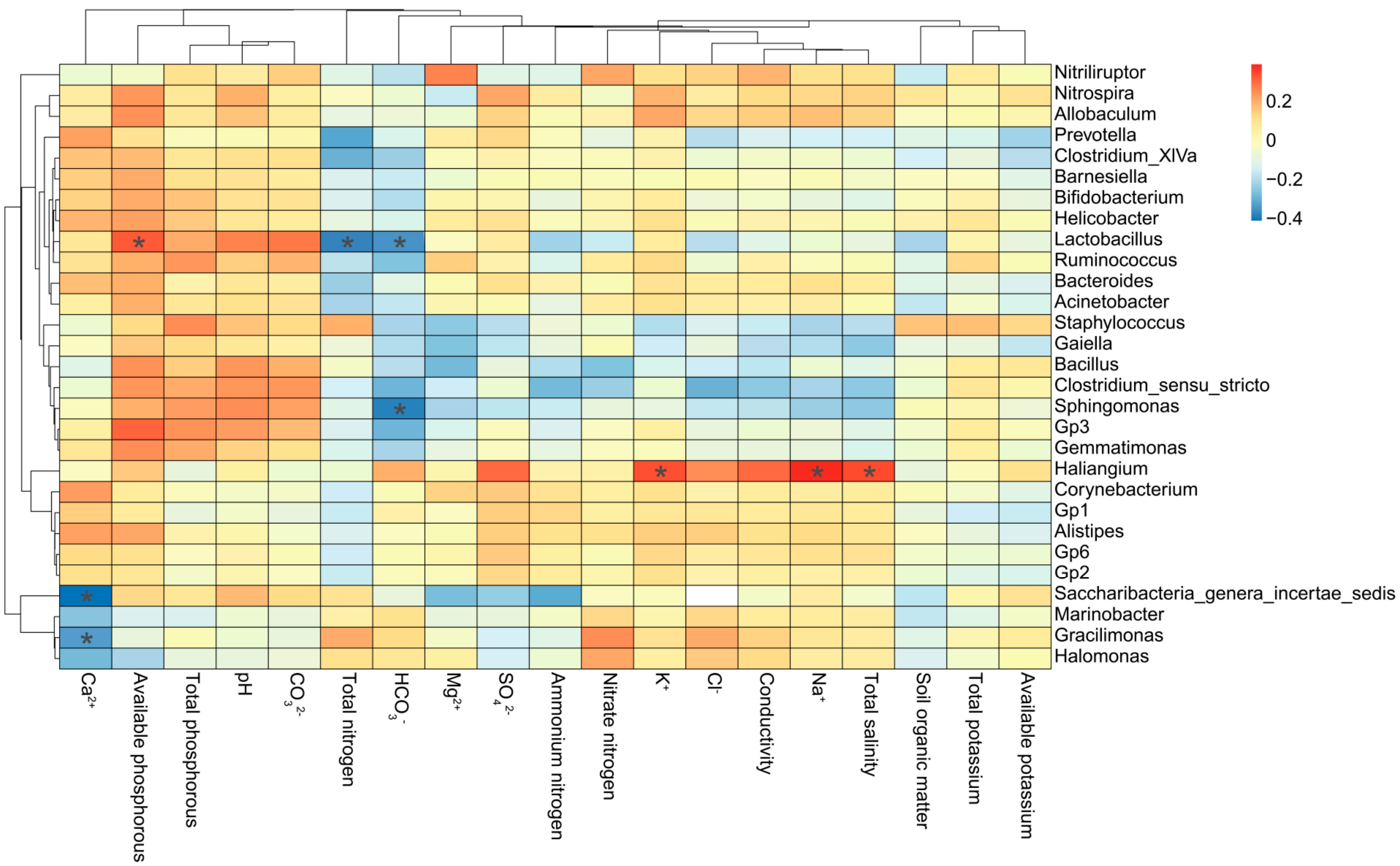

Supplementary Figure S8: Heatmap of the correlation between the top thirty genera and soil environmental factors (An asterisk indicates a significant correlation)

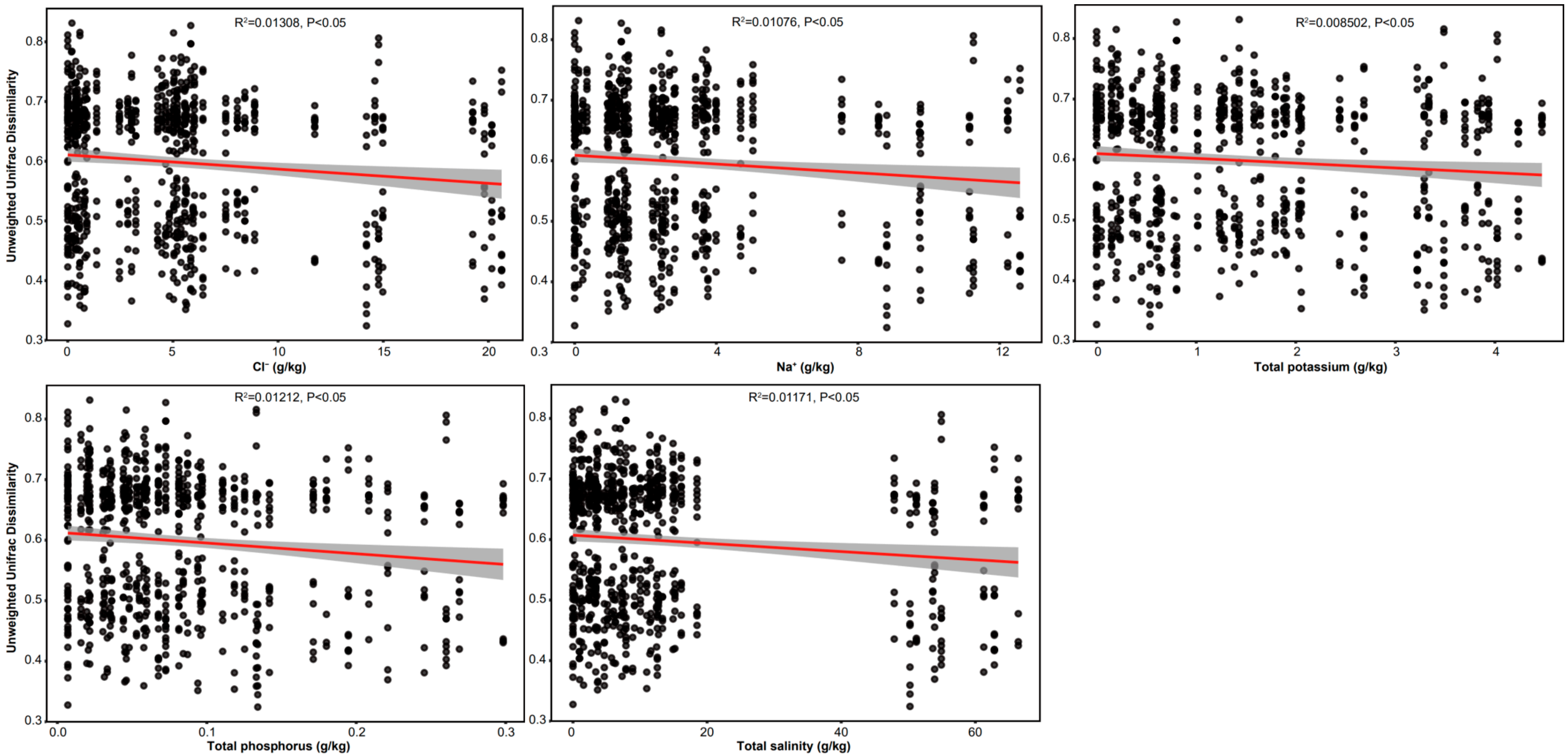

Supplementary Figure S9: Linear regression analysis showing correlations between the bacterial community dissimilarities and difference in the Cl^-, Na+, Total potassium, Total phosphorus, and Total salinity of the studied soil samples (Red shade indicates the 95% confidence).

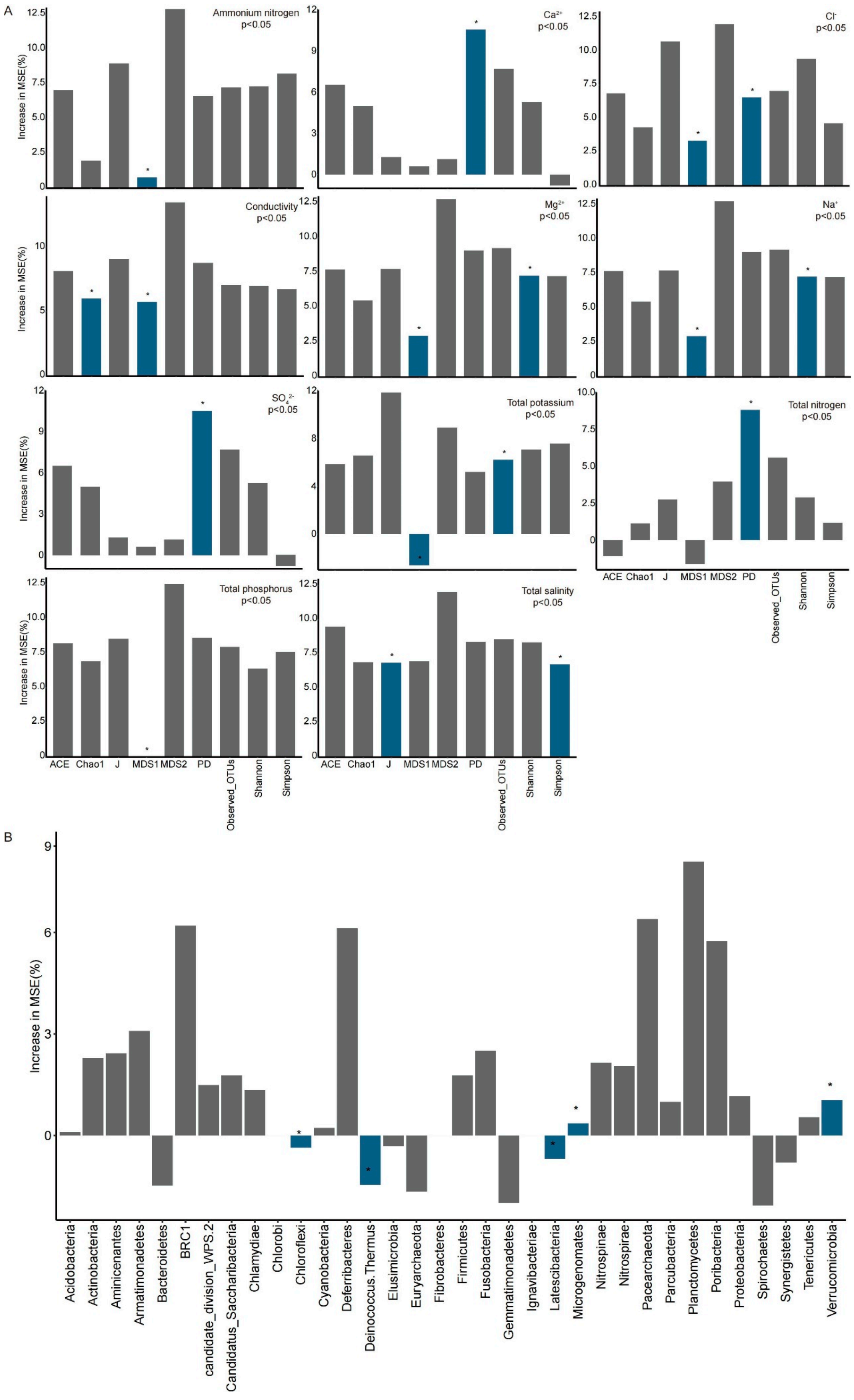
A
Ammonium nitrogen p<0.05
Ca2+ p<0.05
Cl- p<0.05
Conductivity p<0.05
Mg2+ p<0.05
Na+ p<0.05
SO42- p<0.05
Total potassium p<0.05
Total nitrogen p<0.05
Total phosphorus p<0.05
Total salinity p<0.05
Increase in MSE(%)
ACE
Chao1
J
MDS1
MDS2
PD
Observed_OTUs
Shannon
Simpson
B
Increase in MSE(%)
Acidobacteria
Actinobacteria
Aminicenantes
Armatimonadetes
Bacteroidetes
BRC1
candidate_division_WPS.2
Candidatus_Saccharibacteria
Chlamydiae
Chlorobi
Chloroflexi
Cyanobacteria
Deferribacteres
Deinococcus.Thermus
Elusimicrobia
Euryarchaeota
Fibrobacteres
Firmicutes
Fusobacteria
Gemmatimonadetes
Ignavibacteriae
Latescibacteria
Microgenomates
Nitrospinae
Nitrospirae
Pacearchaeota
Parcubacteria
Planctomycetes
Poribacteria
Proteobacteria
Spirochaetes
Synergistetes
Tenericutes
Verrucomicrobia

Supplementary Figure S10: A, Random Forest (RF) mean predictor importance (percentage of increase of mean square error) of microbial alpha- and beta-diversity indices as drivers for soil properties. B, Random Forest (RF) mean predictor importance (percentage of increase of mean square error) of all microbial phyla as drivers for Na^{+}. The accuracy importance measure was computed for each tree and averaged over the forest (5000 trees). Percentage increases in the MSE (mean squared error) of variables were used to estimate the importance of these predictors, and higher MSE% values imply more important predictors. Significance levels are as follows: *, $P < 0.05$; MSE, mean squared error.
